# Supplementary material for: Optical wireless link between a nanoscale antenna and a transducing rectenna
Source: Nat Commun. 2018 May 18;9:1992. doi: 10.1038/s41467-018-04382-7 (PMC5959908; doi:10.1038/s41467-018-04382-7)
Supplement: Supplementary file 1 — Supplementary Information [file 41467_2018_4382_MOESM1_ESM.pdf]

Supplementary Information

Optical wireless link between a nanoscale antenna and a transducing rectenna

Dasgupta et al.

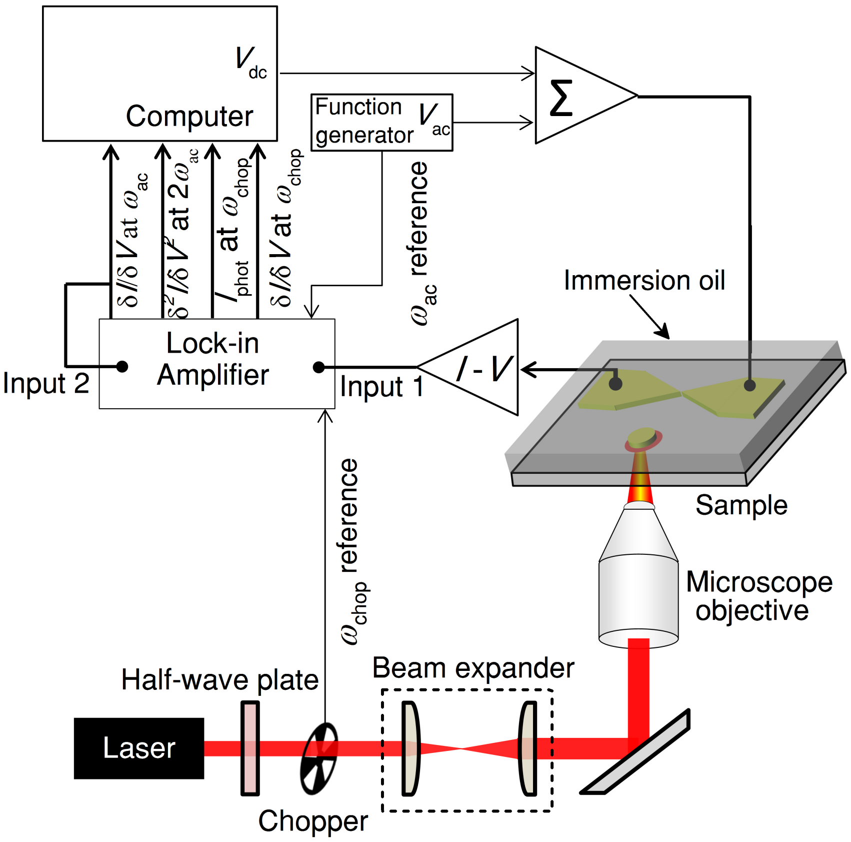

Supplementary Figure 1| Schematic representation of the experimental arrangement for optical and electrical characterization.

Supplementary Note 1.

Gap size estimation using Simmons' model

We estimate the gap width of the electromigrated tunnel junction by analyzing the experimentally recorded nonlinear I-V characteristics using the general expression provided by John G. Simmons to calculate the tunneling current through metal-insulator-metal (MIM) system.[1,2] Supplementary Figure

2a is an energy diagram illustrating electron tunneling through a MIM junction polarized by a bias V_{dc}. Here, ϕ_1 , ϕ_2 are the energy barrier heights of the metal on the left side and the right side of the MIM interface and E_F is the Fermi energy. Following the interpretation provided by Brinkman *et al*, the simplified expression for the tunneling current (I) through such a junction is given by,[3]

$$I = A \times G(0)\left[V_{dc} - \left(\frac{A_0 \Delta\emptyset}{32\emptyset^{3/2}}\right) eV_{dc}^2 + \left(\frac{3A_0{}^2}{128\ \emptyset}\right) e^2 V_{dc}^3\right] \qquad \text{Supplementary Equation 1}$$

Where, $\phi = \dfrac{\phi_1 + \phi_2}{2}$ and $\Delta\phi = \phi_1 - \phi_2$ in eV represent the average barrier height and the asymmetry in the barrier height on both sides of the MIM junction. In Supplementary Equation 1, A is the cross-section of the junction in nm^2, g is the gap width in Å, $A_0 = 4g\dfrac{\sqrt{2m}}{3\hbar}$ and $G(0) = 3.16\times10^{10}\dfrac{\sqrt{\phi}}{g}\exp(-1.025g\sqrt{\phi})$.

In our experiment, we expect a very minimal asymmetry in the barrier height over the gap since the material on both sides is similar. However, a residual asymmetry is generally observed[4,5], which probably results from a geometry-dependent modification of the barrier height[6]. In general, for bulk Au-SiO_2 interface the height of the Schottky barrier is around 4.5 eV as SiO_2 has an electron affinity of 0.75 eV.[7] However, in case of atomic scale gaps, formation of image charges at the metal-insulator interfaces may result in significant lowering of the height of the barrier[8-10]. We include all these aspects into our calculation to estimate the set of parameters g, ϕ and $\Delta\phi$ by fitting the experimentally recorded I-V characteristics curve with Supplementary Equation 1 upon fixing the cross-section area A to a constant value. It is experimentally difficult to infer A as electron microscopy provides a general configuration of the junction but failed to indicate where tunneling is really occurring. Imaging a pristine gap on a non-conductive glass substrate with a high resolution SEM microscope proves to be extremely challenging. This precludes operating the SEM under optimal acceleration voltages because charging effects will inevitably disturb the image. In the SEM micrograph presented in the manuscript,

the image was obtained by sputtering the device with a thin layer of Au to enable the evacuation of the charges. The procedure is thus destructive.

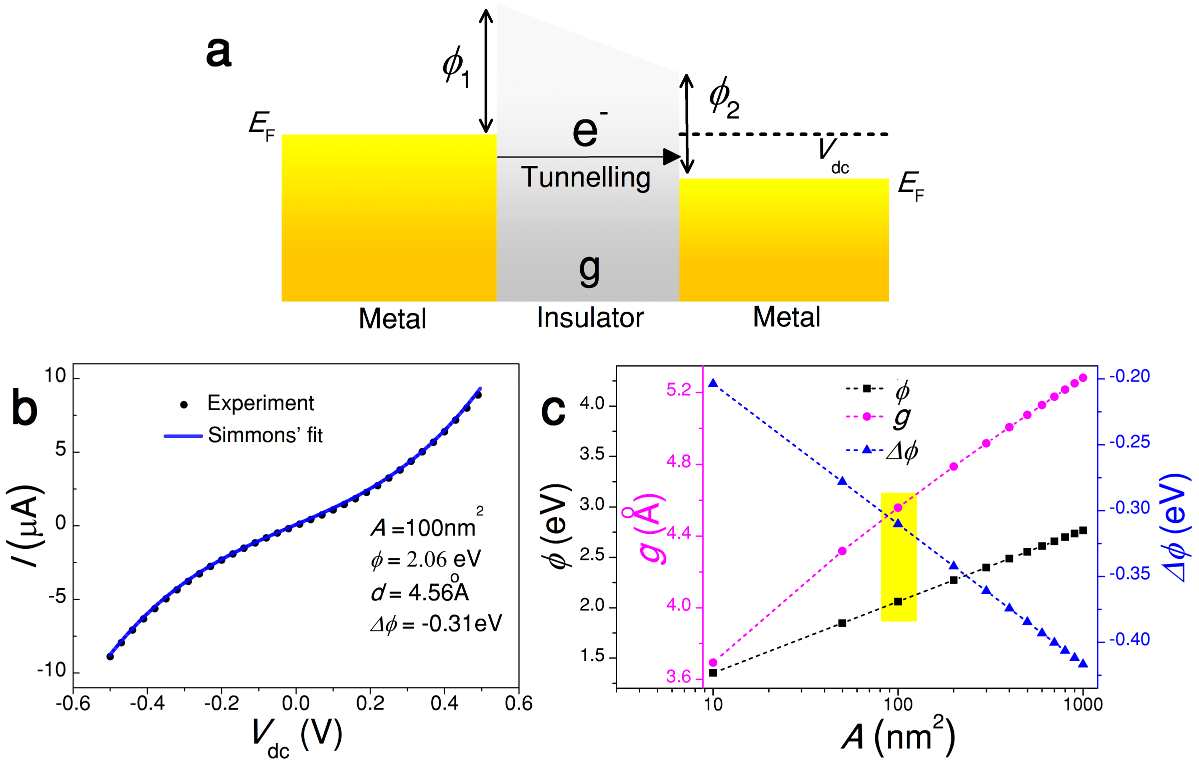

Supplementary Figure 2l Estimation of the electromigrated junction gap width. a Energy diagram illustrating the electron tunneling through an atomic scale MIM junction under an applied bias V_{dc}. **b** I-V characteristics of the electromigrated junction used in our experiment. The black data points represent the experimentally measured I-V characteristics of the junction. The blue plot is the Simmons' formula fit of the experimental data with fitting parameters A = 100 nm^2, ϕ = 2.06 eV, $\Delta\phi$ = -0.31 eV and g = 4.56 Å. **c** Evolution of all the fitting parameters (ϕ, $\Delta\phi$ and g) as a function of cross section area A. The points highlighted by yellow color indicate the set of parameters obtained from the fit represented in Supplementary Figure 2b.

Supplementary Figure 2b shows the fitting (red line) of the experimentally recorded I-V plot (black data points) assuming a cross-section of 100 nm^2 (an active area which is 10 nm thick by 10 nm wide) which results in an estimation of the gap width g = 4.56 Å with ϕ = 2.06 eV and $\Delta\phi$ = -0.31eV. In Supplementary Figure 2c, we plot the evolution of all the parameters as a function of various cross-sectional areas. From these calculations, we conclude that the tunneling gap has an effective width remaining below 5 Å.

A study by Frimmer et al. suggested that the Ti adhesion layer evaporated between the glass substrate and the Au may form a TiO_2 barrier at the electromigrated gap. Based upon the representation of the

IV characteristics in the form of a Fowler-Nordheim plot and an analysis of the transition voltage, the authors proposed a modified energy barrier and concluded that the tunneling transport occurs through TiO_2[11]. However, interpretation of transition voltage spectroscopy as a measure of the barrier height is highly debated in the literature. In fact, recent studies suggest that transition voltage observed in Fowler-Nordheim plots do not stand for a change in the electron transport mechanism from direct tunneling to field emission. Instead, the minimum of the Fowler-Nordheim plot mainly signifies a transition towards a higher nonlinearity order in the I-V curve[12,13]. From the tunneling current expression in Supplementary Equation 1 an analytical expression for transition voltage V_T can be determined:

$$V_T = \frac{2\hbar}{e\sqrt{m}} \frac{\sqrt{3\emptyset}}{g} \qquad \text{Supplementary Equation 2}$$

where m is the mass of electron, e is the charge of electron and g is the gap width in Angstrom. The Supplementary Equation 2 indicates that V_T is not only related to the barrier height (ϕ) but also to the gap size (g). For the sake of illustration, we analyze the Fowler-Nordheim plot of another electromigrated junction prepared under the same experimental condition. The zero bias conductance is here G = 2.2 μS (0.028 G_0). We display the Simmons' fit (blue plot) of the experimentally recorded I-V characteristics (black data points) in Supplementary Figure 3a. The fit leads to an estimation of g = 4.81Å with ϕ = 3.38 eV and $\Delta\phi$ = 1.22 eV under the assumption of the cross- sectional area of the gap being 100 nm^2.

We show in Supplementary Figure 3b the same experimental data points represented in a Fowler-Nordheim graph. This type of representation highlights two regimes separated by the inflection points of the curve traditionally referred as the transition voltages. Now by inserting g, ϕ, and $\Delta\phi$ into the Supplementary Equation 2, we predict a transition voltage to a higher-order nonlinearity at around V_T = 0.92 V. This is very close to the average transition voltage deduced from the graph in Supplementary Figure 3b at V_T = 0.88 eV. Because of the dependence of V_T on $\frac{\sqrt{\phi}}{g}$ and not only ϕ, we

believe that an interpretation of the energy barrier based upon the Fowler-Nordheim graph is not sufficient to determine the role of a TiO_2 layer.

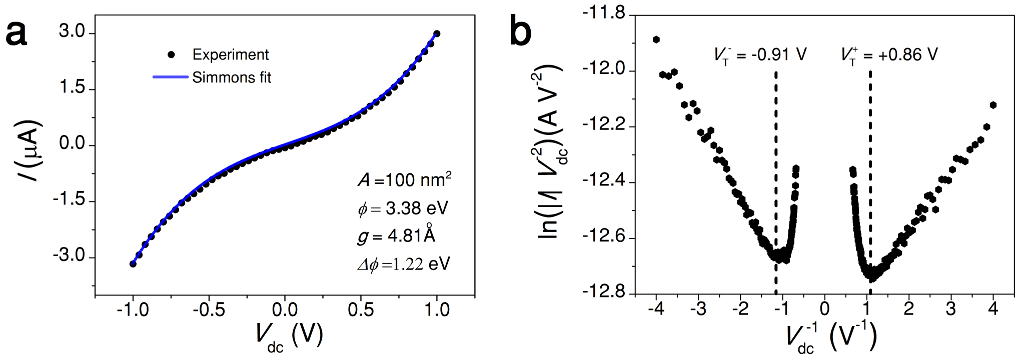

Supplementary Figure 3l Analysis based on Fowler-Nordheim representation of I-V characteristics. a, nonlinear I-V curve for another electromigrated junction for which we again estimate the gap width. Black data points are the experimentally measured currents and the blue plot is the Simmons' fit from which we estimate the gap-width of this particular junction to be g = 4.56 Å. **b,** The Fowler-Nordheim plot for the same junction which indicates transition voltages around V_T^+ = 0.86V and V_T^- = -0.91V.

Supplementary Note 2.

Characterization of thermal contributions in optically rectified current. As discussed in the main article, optical rectification process takes place when the feed-gap receives radiation, either by a direct illumination or via a distant transmitting antenna. When the junction is directly illuminated, the photocurrent can be largely dominated by laser-induced thermal effects *i.e.*, thermal expansion of the metallic electrical leads forming the gap and building up of thermo-voltage[14,15] as well as tunneling from photo-excited carriers crossing the barrier height[16]. Laser-induced thermal contributions are expected when the metallic electrodes absorb part of the incoming energy flux and may be observed in the I_{phot} map[4,14] even if the feed-gap is outside the excitation area.

First, let us consider a laser-induced expansion of the Au electrodes resulting in a reduction of the gap width. The rise in the electrical conductance of the device leads to an increased current flowing through the circuit whenever the laser is positioned on the metallic electrodes. The exact contribution depends on the absorption cross-section of the electrode receiving the incoming light. Second, the

absorption of the laser by either of the electrodes creates a temperature gradient across the feed gap leading to a built-up thermo-voltage. However, when the gap is symmetrically illuminated with a centered laser beam, the temperature on both sides is approximately the same and the thermoelectric response is mitigated. Therefore, the combined effects of electrode expansion and thermo-voltage should be predominant in the I_{phot} map when the laser is positioned on the metal electrode away from the junction.

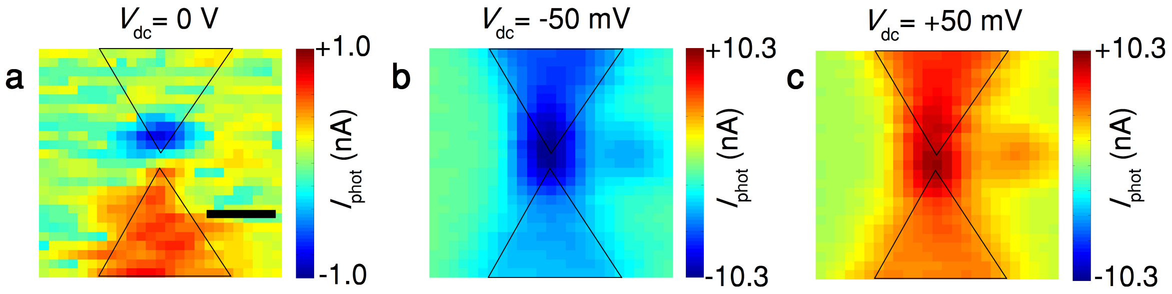

Supplementary Figure 4| Photocurrent map for direct exposure of the electromigrated junction. a, I_{phot} map for a zero applied dc bias (V_{dc} = 0 V). There is no sign of rectification when the laser is focused on the junction but thermally induced current is visible when laser is focused on one of the electrodes away from the junction. Scale bar is 1 μm. **b,** and **c,** are I_{phot} maps of the same area for an applied d.c. bias of V_{dc} = -50 mV and +50 mV, respectively. Optical rectification is apparent when the laser is focused exactly on the gap. The rectified current follows the sign of the applied d.c. bias. The polarization of the incident field is vertical.

Direct illumination of the feed-gap. To confirm that the measured photocurrent is created through an optical rectification process, we perform a series of experiments to characterize and rule out these thermal effects. First, we record a photocurrent map for an incident laser intensity of 353 kW cm^{-2} by scanning the feed-gap through the focused spot at an applied bias V_{dc} = 0 V. As shown in the photocurrent map of Supplementary Figure 4a, we observe approximately zero photocurrent when the laser is exactly focused on the gap. This is expected as at V_{dc} = 0 V, the nonlinearity of the conductance is small and the rectification is thus minimal [$I_{phot}=1/4V_{opt}^2(\partial^2 I/\ \partial V^2)$] as discussed in main article. Also absence of a response in I_{phot} at the gap suggests that any thermal expansion of the electrodes can be ruled out. Because the metal electrodes are physically deposited on the glass surface, thermal expansion is consequently negligible. Furthermore, the tapered geometry of the electrodes acts

as a heat sink and is thus more efficient at dissipating the absorbed energy. In the light of the small but measurable asymmetry of the I-V characteristics, the absence of photocurrent when the feed-gap is illuminated also suggests that tunneling of photo-excited carriers above the energy barrier is an unlikely process contributing to the photocurrent. Supplementary Figure 4a shows an inversion of the sign of I_{phot} when the focused laser irradiates the top or bottom electrode. This inversion of the contrast is a clear signature of a thermo-voltage developing across the gap[4,14,17]. The signed current is also present in the I_{phot} maps shown in Supplementary Figure 4b and c where applied biases are -50 mV and +50 mV, respectively. For nonzero applied biases, we observe an enhancement in I_{phot} response when the laser is positioned on the gap.

Recently, Zolotavin et al. suggested that exciting the constriction between the large electrodes at plasmon resonance leads to formation of long lived hot carriers populated at the gap which results in very large photo-thermal voltages at the unbiased junction[15]. In their work, they observed this effect when the constriction was selectively excited at its transverse plasmon resonance (excitation polarization perpendicular to the orientation of the electrodes). If this phenomenon is present, a large photocurrent flowing through the device should be observed when the laser is positioned on the nano-junction, even at V_{dc} = 0 V. The photocurrent map for V_{dc} = 0 V shown in Supplementary Figure 4a makes clear that such response is not occurring here. This is expected considering the excitation condition and the geometry of the device. In the present scenario, the 785 nm wavelength laser is polarized along the orientation of the electrodes and is thus enable to resonantly excite the plasmon response of the 100 nm wide constriction.

From this set of data, we conclude that when the junction is illuminated symmetrically with a centered laser beam, optical rectification is predominant and the recorded photocurrent is devoid of any laser-induced thermal contributions.

Illumination of the transmitter antennas. To complement the above experiment we monitor the conductance $\partial I/\partial V$ at f_{chop} when adjacent nanoantennas are illuminated. Laser-induced thermal variation of the conductance should modulate the recorded photocurrent[14]. We simultaneously map the I_{phot} signal and $\partial I/\partial V$, both at f_{chop} by scanning the laser through the area comprising the optical

antennas as presented in Supplementary Figure 5a and b, respectively. The incident polarization of the laser is kept along the vertical axis for the entire experiment (maximized antenna transmission towards the rectenna). It is evident from these maps that we could not measure a change even down to 10^{-5} level in the conductance map which can be correlated to the recorded I_{phot} signal. Therefore, we can infer from this experiment that the recorded photocurrent is produced through the optical rectification of the transmitted radiation and not due to any laser-induced modulation of the junction conductance.

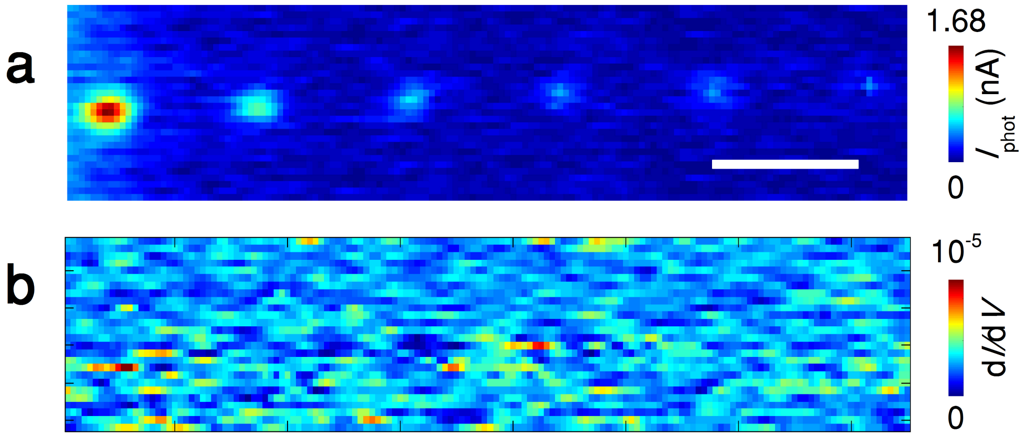

Supplementary Figure 5 I a, I_{phot} map presented in Fig. 2a of the main text. Scale bar is 2 μm. **b,** Simultaneously acquired map of *dI/dV* demodulated at the frequency of the optical chopper. No measureable contrast in this image can be related to the I_{phot} map. This indicates that the photocurrent is free of any laser-induced conductance change of the feed-gap.

Supplementary Note 3.

V_{opt} vs incident polarization measurement. To measure the optically induced a.c. voltage drop V_{ac} at the gap, we focus the laser on an individual optical antenna located 4 μm away from the junction and simultaneously monitor I'' and I_{phot} as a function of applied bias V_{dc} while rotating the incident polarization from 0° to 90°. The laser intensity is kept at 540 kW cm^{-2} for the whole experiment. For each incident polarization, V_{ac} is adjusted in such a way so that I_{phot} follows I'' for the entire V_{dc} sweep. This is only possible when the optical rectification is the main mechanism behind I_{phot}. The results are

plotted in Supplementary Figure 6. Here data points represent the amplitude of I_{phot} and the lines are I'' for the corresponding cases. The value of V_{ac} for which we obtain $I_{phot}=I''$ is recorded as the V_{opt} in the main article.

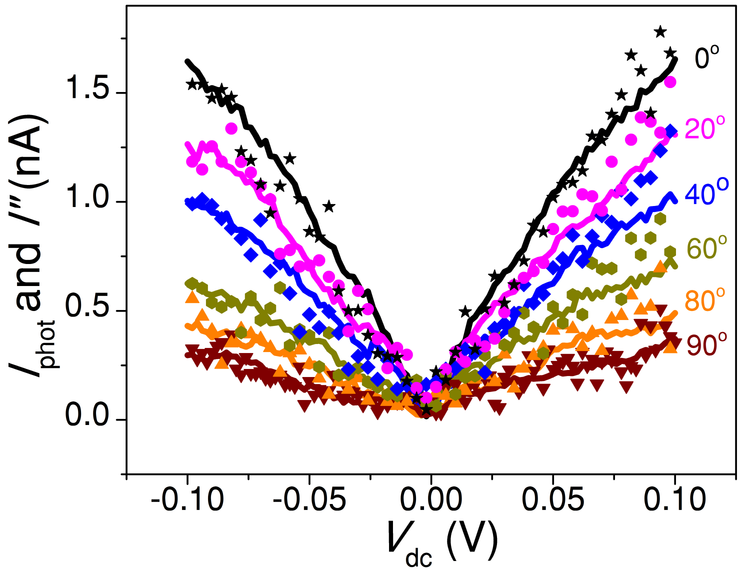

Supplementary Figure 6l Polarization measurement. Evolution of I_{phot} and I'' for a V_{dc} sweep for different incident polarizations and a laser intensity of 540 kW cm^{-2}. The data points represent I_{phot} and the lines represent I'' in each plot. For each polarization, V_{ac} is adjusted so that I_{phot} and I'' are of same amplitude. The value of V_{ac} for conditioning $I_{phot}=I''$ is recorded as V_{opt} in the main article.

Supplementary Note 4.

Reproducibility. In the inset of Supplementary Figure 7, we show a I_{phot} map of an area comprising transmitting antennas of a second tested device. The electrical characteristics of this second rectenna are shown in Supplementary Figure 3a. The map is obtained for an incident laser intensity of 707 kW cm^{-2} under an applied bias of V_{dc} = 100 mV across the junction. Very much like the device discussed in the main text, the plot in Supplementary Figure 7 shows that the I_{phot} response follows the similar generic power law dependence to the distance separating the rectenna to the transmitting antennas. The data points in the plot are the experimentally obtained values for excitation of each antennas and the solid black curve represents the power law fit.

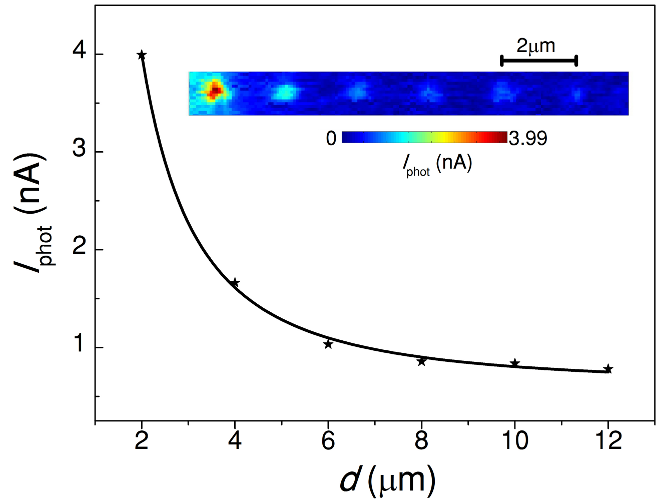

Supplementary Figure 7| Demonstration of optical wireless link for a second tested device. The data points are the photocurrent generated by the rectenna for antennas located at increasing distances. The data are extracted from the photocurrent map displayed in the inset. The generic power law ($\alpha d^{b}+c$) fit (solid line) converges at an exponent value of b = 1.77. The I-V characteristics of the device is presented in Supplementary Figure 3a.

The reproducibility and the device-to-device variability for the broadcasting scheme presented in our work mainly depend on the electrical properties of the electromigrated junctions. In Supplementary Table 1, we present a statistics of the zero-bias conductances of 24 electromigrated junctions. As shown in the table, 17% of the junctions feature an electrical conductance comparable to the rectenna reported in the main article.

Zero bias Conductance G (S)	Percentage of electromigrated junctions (%)
$10^{-1}G_0<G<G_0$	16.7
$10^{-2}G_0<G<10^{-1}G_0$	8.3
$10^{-3}G_0<G<10^{-2}G_0$	12.5
$10^{-4}G_0<G<10^{-3}G_0$	25.0
$10^{-5}G_0<G<10^{-4}G_0$	4.1
$G<10^{-5}G_0$	33.3

Supplementary Table 1 Statistics of the zero bias tunneling conductance for a set of 24 electromigrated junctions

Supplementary Note 5.

Effect of the size of the antennas on the scattering of the incoming radiation. To support the numerical calculations presented in Fig. 4b of the main article we perform numerical studies presented in Supplementary Figure 8. In the main text, we show that using a 110 nm diameter antenna as a transmitter instead of a 220 nm unit results in a lower electromagnetic field at the junction (Fig. 4b). Here, we are interested at numerically investigating the role of the antenna diameter on defining what amount of the incident radiation is effectively scattered from it. For this, we perform a 3D-FEM simulation (COMSOL Multiphysics) considering a 785 nm wavelength, focused Gaussian excitation of 600 nm diameter spot size. This corresponds to a full-width-at-half-maximum of ~350 nm, a value closed to the point-spread function of the objective. We calculate the scattering efficiency (in percentage of total incident power) by varying the diameter of the antenna for an incident polarization perpendicular to the preferred transmitting axis (i.e. towards the rectenna). For each diameter, the calculated total power of the scattered radiation is normalized to the total incident power.

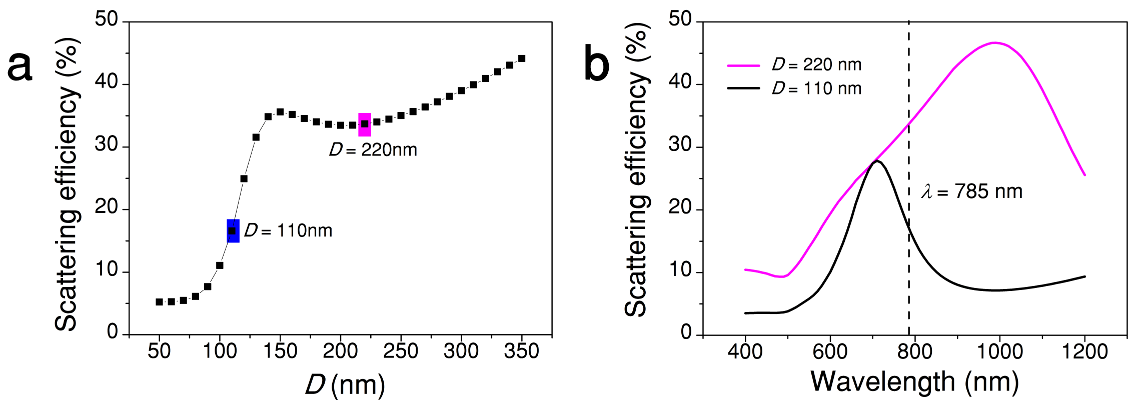

Supplementary Figure 8 | Size dependence of the scattering coefficients of the optical nanoantennas. a, Variation of the antenna' scattering efficiency as a function of the diameter *D*. **b,** Scattering spectra for antenna diameter *D* = 110 nm (black plot) and *D* = 220 nm (red plot). All calculations are obtained considering a 600 nm diameter Gaussian excitation.

Supplementary Figure 8a shows the variation of the scattering efficiency as a function of antenna diameter. The shoulder at diameter ~150 nm indicates the resonant condition. Comparing quantitatively the scattering efficiency, the graph shows a larger value for a 220 nm antenna (34%)

compared to the scattering efficiency for a 110 nm antenna (16%). Note that the efficiency on resonance is not significantly higher, because the scattering efficiency scales with D^6. The amount of signal scattered towards the rectenna increases with antenna diameter and explains the higher electric field at the feed-gap for a 220 nm transmitting antenna (Fig. 4b in the main text). In Supplementary Figure 8b, we plot the scattering spectra for the 110 nm antenna (black plot) and the 220 nm antenna (magenta plot). For both antennas the excitation is located on the shoulder of the dipolar surface plasmon resonance, explaining thus the polarization sensitivity of the wireless link.

Supplementary Note 6.

Effect of the presence of other antennas in the line-of-sight. In this section, we numerically estimate the effect of the presence of other antennas in the path of transmission towards the rectenna on the field localization at the rectenna feed-gap. For that, we place five antennas at incremental distances from the rectenna with a step size of 2 μm. We calculate the normalized electric field at the junction by illuminating one of these antennas at a time with the vertical incident polarization. In Supplementary Figure 9a, we illustrate the calculated field distribution around the structures in logarithmic scale when an optical antenna located 6 μm away from the feed-gap is excited. It is clear that the presence of the nanoparticles induces additional scattering of the field radiated from the excited element. In the plot of supplementary Figure 9b, we compare the calculated electric field values (black data points) with the values (magenta plot) determined when the calculations do not include other discs in the line of sight (Fig. 4b of the main article). The electric field decreases slightly and at maximum 16% reduction is observed for the antenna located 10 μm away from the junction. The transmitted radiation is thus shadowed due to the presence of scattering elements in the path to the rectenna, but the effect remains minimal in this scenario.

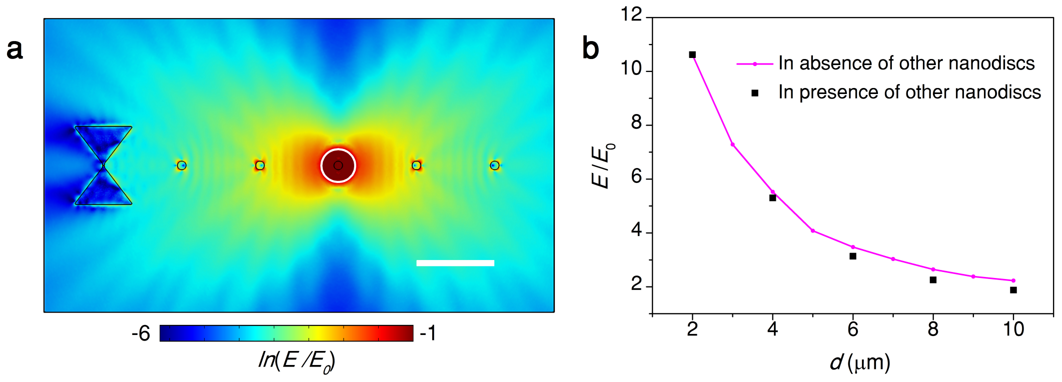

Supplementary Figure 9 | Effect of the presence of adjacent antennas in the path of transmission. a, Calculated normalized electric field distribution around the nanostructures when a nanodisc at a distance of 6 μm is excited in presence of other nanodiscs in the transmission path. Scale bar is 2 μm. **b,** The magenta plot represents the electric field values at the location of the rectenna feedgap derived when the calculation does not include any antennas placed in line-of-sight. The black data points represents the calculated electric field values when 5 nanodiscs are always present in the simulation geometry and each of them is excited individually.

Supplementary Note 7

Comparison with direct illumination of the nano-gap. For the sake of completion of our study, we also compared the difference between the rectified current produced through direct illumination of the gap and through broadcasted signal from the adjacent nano-antennas. The values for each case for an illumination intensity of 353 kW cm^{-2} and an applied bias of V_{dc} = 50 mV are shown in Supplementary Table 2.

Illumination point	Rectified current (nA)
Direct	10.3
Antenna 1 (2μm)	1.67
Antenna 2 (4μm)	0.74
Antenna 3 (6μm)	0.58
Antenna 4 (8μm)	0.49
Antenna 5 (10μm)	0.45
Antenna 6 (12μm)	0.43

Supplementary Table S2: Direct rectification vs transduction of the broadcasted signal

Supplementary References

1 Simmons, J. G. Generalized formula for the electric tunnel effect between similar electrodes separated by a thin insulating film. *Journal of Applied physics* **34**, 1793-1803 (1963).

2 Simmons, J. G. Electric tunnel effect between dissimilar electrodes separated by a thin insulating film. *Journal of Applied physics* **34**, 2581-2590 (1963).

3 Brinkman, W., Dynes, R. & Rowell, J. Tunneling conductance of asymmetrical barriers. *Journal of Applied physics* **41**, 1915-1921 (1970).

4 Stolz, A. *et al.* Nonlinear photon-assisted tunneling transport in optical gap antennas. *Nano Letters* **14**, 2330-2338 (2014).

5 Buret, M. *et al.* Spontaneous hot-electron light emission from electron-fed optical antennas. *Nano Letters* **15**, 5811-5818 (2015).

6 Mayer, A. *et al.* Analysis of the efficiency with which geometrically asymmetric metal–vacuum–metal junctions can be used for the rectification of infrared and optical radiations. *Journal of Vacuum Science & Technology B, Nanotechnology and Microelectronics: Materials, Processing, Measurement, and Phenomena* **30**, 031802 (2012).

7 Nobuyuki, F., Akio, O., Katsunori, M. & Seiichi, M. Evaluation of valence band top and electron affinity of SiO 2 and Si-based semiconductors using X-ray photoelectron spectroscopy. *Japanese Journal of Applied Physics* **55**, 08PC06 (2016).

8 Binnig, G., Garcia, N., Rohrer, H., Soler, J. & Flores, F. Electron-metal-surface interaction potential with vacuum tunneling: Observation of the image force. *Physical Review B* **30**, 4816 (1984).

9 Ma, X., Shu, Q., Meng, S. & Ma, W. Image force effects on trapezoidal barrier parameters in metal–insulator–metal tunnel junctions. *Thin solid films* **436**, 292-297 (2003).

10 Nguyen, H., Q., Feuchtwang, T., E. & Cutler, P., H. Do Tunneling Electrons Probe The Image Interaction ? *J. Phys. Colloques* **47**, C2-37-C32-44 (1986).

11 Frimmer, M., Puebla-Hellmann, G., Wallraff, A. & Novotny, L. The role of titanium in electromigrated tunnel junctions. *Applied Physics Letters* **105**, 221118 (2014).

12 Vilan, A., Cahen, D. & Kraisler, E. Rethinking transition voltage spectroscopy within a generic Taylor expansion view. *ACS Nano* **7**, 695-706 (2012).

13 Huisman, E. H., Guédon, C. M., van Wees, B. J. & van der Molen, S. J. Interpretation of transition voltage spectroscopy. *Nano Letters* **9**, 3909-3913 (2009).

14 Ward, D. R., Hüser, F., Pauly, F., Cuevas, J. C. & Natelson, D. Optical rectification and field enhancement in a plasmonic nanogap. *Nature Nanotechnology* **5**, 732-736 (2010).

15 Zolotavin, P., Evans, C. & Natelson, D. Photothermoelectric effects and large photovoltages in plasmonic Au nanowires with nanogaps. *The Journal of Physical Chemistry Letters* **8**, 1739-1744 (2017).

16 Diesing, D., Merschdorf, M., Thon, A. & Pfeiffer, W. Identification of multiphoton induced photocurrents in metal–insulator–metal junctions. *Applied Physics B* **78**, 443-446 (2004).

17 Xu, X., Gabor, N. M., Alden, J. S., van der Zande, A. M. & McEuen, P. L. Photo-thermoelectric effect at a graphene interface junction. *Nano Letters* **10**, 562-566 (2009).
